# Coagulation and Inflammatory Responses After Tourniquet Release in Total Knee Arthroplasty: Association with Hemodynamic Instability

**DOI:** 10.3390/jcm15145386

**Published:** 2026-07-09

**Authors:** Fatma Acil, Cemal Nas, Andaç Dedeoğlu, Hülya Tosun Söner, Ali İhsan Yürekli, Kutbettin Dinçer, Cahit Ancar, Erhan Gökçek, Abdulkadir Yektaş

**Affiliations:** 1 Department of Anesthesiology and Reanimation, Diyarbakır SBÜ Gazi Yaşargil Training and Research Hospital, Diyarbakır 21010, Turkey; anderen77@hotmail.com (A.D.); hulyatosunsoner@hotmail.com (H.T.S.); dryurekli06@gmail.com (A.İ.Y.); gokcekerhan_44@hotmail.com (E.G.); akyektas@hotmail.com (A.Y.); 2 Department of Biochemistry, Diyarbakır SBÜ Gazi Yaşargil Training and Research Hospital, Diyarbakır 21010, Turkey; drcemalnas@gmail.com; 3 Department of Orthopedics and Traumatology, Diyarbakır SBÜ Gazi Yaşargil Training and Research Hospital, Diyarbakır 21010, Turkey; baran_06fb@hotmail.com (K.D.); dr_cahit2006@hotmail.com (C.A.)

**Keywords:** total knee arthroplasty, tourniquet release, hemodynamic instability, coagulation, inflammation

## Abstract

**Background:** Tourniquet use during total knee arthroplasty (TKA) improves surgical visualization and limits blood loss but may also trigger ischemia–reperfusion-related hemodynamic instability. This study investigated coagulation and inflammatory responses after tourniquet release and their association with perioperative hemodynamic instability. **Methods**: This prospective observational cohort study included 22 patients aged 65–90 years undergoing unilateral TKA. Hemodynamic parameters, coagulation markers (D-dimer, INR, APTT), and inflammatory markers were measured at predefined perioperative time points. Hemodynamic instability was assessed using the modified shock index (MSI = HR/MAP). Non-parametric statistical analyses were performed. **Results:** The D-dimer and INR increased significantly after tourniquet release and remained elevated at 24 h (*p* < 0.001). APTT prolongation was transient (*p* = 0.001). The NLR and SII increased both early and late, whereas CRP showed a delayed rise. Δ analysis demonstrated temporal changes in coagulation parameters; however, no significant between-group differences according to MSI status were identified. Although ΔPeak IL-6 was higher in the unstable group, the difference was not statistically significant (*p* = 0.097). ROC analysis demonstrated moderate discriminative performance for ΔPeak IL-6 (AUC = 0.747), whereas the NLR (AUC = 0.471), SII (AUC = 0.529), and CAI (AUC = 0.482) showed poor predictive performance for hemodynamic instability. **Conclusions:** Tourniquet release induces significant coagulation and inflammatory responses following total knee arthroplasty. Although no definitive biomarker of hemodynamic instability was identified, ΔPeak IL-6 showed a trend toward association with instability and may merit further investigation. These findings suggest a possible interaction between inflammatory and coagulation pathways after tourniquet release and should be validated in larger prospective studies.

## 1. Introduction

Total knee arthroplasty (TKA) is one of the most commonly performed orthopedic procedures worldwide and remains the definitive treatment for end-stage knee osteoarthritis. With the aging population and increasing prevalence of degenerative joint disease, the number of TKA procedures continues to rise. Despite substantial improvements in surgical techniques and perioperative care, postoperative complications related to thromboembolic events, inflammatory responses, and hemodynamic instability remain important causes of morbidity and prolonged hospitalization. Therefore, a better understanding of the physiological responses triggered during TKA is essential for optimizing perioperative management and improving patient outcomes [1,2]. Among these challenges, maintaining hemodynamic stability after tourniquet release remains an important but incompletely understood aspect of perioperative care.

Among these physiological responses, the hemodynamic consequences of tourniquet release have received increasing attention because of their potential impact on perioperative safety and recovery. Hemodynamic instability following tourniquet release represents a particularly important concern in elderly patients undergoing TKA, as abrupt reperfusion may trigger complex cardiovascular, inflammatory, and coagulation-related responses that can adversely affect perioperative outcomes [3,4].

Traditional perioperative monitoring relies primarily on conventional hemodynamic variables such as heart rate and arterial blood pressure. However, these parameters may not fully reflect the complex physiological alterations occurring immediately after tourniquet release. Composite hemodynamic indices, such as the modified shock index (MSI), integrate both heart rate and perfusion pressure and may provide a more sensitive assessment of early circulatory instability during the reperfusion phase following tourniquet release [5].

These hemodynamic alterations are largely initiated by ischemia–reperfusion injury associated with tourniquet application. During TKA, tourniquets are commonly applied to provide a clearer operative field and to help limit perioperative bleeding. In addition, previous studies have reported that tourniquet use may shorten operative time [6,7].

Despite these advantages, tourniquet inflation and subsequent deflation may induce a systemic inflammatory response and ischemia–reperfusion injury [8]. Reperfusion initiates an early inflammatory response characterized by increased TNF-α and IL-1 activity, followed by broader activation of downstream inflammatory mediators over the subsequent 4–48 h [8,9]. This is accompanied by activation of anti-inflammatory pathways, including interleukin-10 (IL-10) [10,11].

Beyond its local effects on the operative field, tourniquet inflation and subsequent deflation may substantially influence systemic coagulation pathways. Prolonged limb ischemia followed by reperfusion can activate endothelial cells, platelets, and leukocytes, resulting in the release of procoagulant mediators and inflammatory cytokines [3,4,12]. This process may promote thrombin generation, enhance fibrin formation, impair endogenous fibrinolysis, and contribute to a transient hypercoagulable state. Previous studies have suggested that tourniquet use during TKA may influence postoperative coagulation activity and contribute to perioperative complications, including thromboembolic events and delayed recovery. In addition, alterations in fibrinolytic activity, thrombin generation, and endothelial function have been reported following tourniquet-assisted orthopedic procedures [12].

Understanding this complex inflammatory response is crucial, as it may contribute to clinically relevant hemodynamic disturbances, including hypotension, bradycardia, impaired tissue perfusion, and transient circulatory instability following tourniquet release. These events likely reflect a multifactorial process involving coagulation activation, endothelial dysfunction, and autonomic imbalance [13,14,15].

Although previous studies have separately investigated inflammatory responses, coagulation activation, and tourniquet-related complications after TKA, studies integrating these biological pathways with perioperative hemodynamic monitoring remain scarce, and their potential contribution to perioperative hemodynamic instability remains poorly characterized. A better understanding of these biological mechanisms may facilitate perioperative risk stratification and support the development of more individualized hemodynamic monitoring strategies in patients undergoing TKA. We hypothesized that tourniquet release during TKA would induce measurable coagulation and inflammatory responses and that the magnitude of these responses would be associated with perioperative hemodynamic instability. Therefore, the present study aimed to evaluate temporal changes in coagulation and inflammatory biomarkers following tourniquet release and to investigate their relationship with hemodynamic instability assessed using the modified shock index (MSI), a hemodynamic parameter previously associated with adverse clinical outcomes and mortality in acute care settings. By integrating coagulation, inflammatory, and hemodynamic data, we sought to provide a more comprehensive understanding of the biological mechanisms underlying perioperative instability after TKA [5].

## 2. Materials and Methods

### 2.1. Study Design and Patients

**Figure 1:**
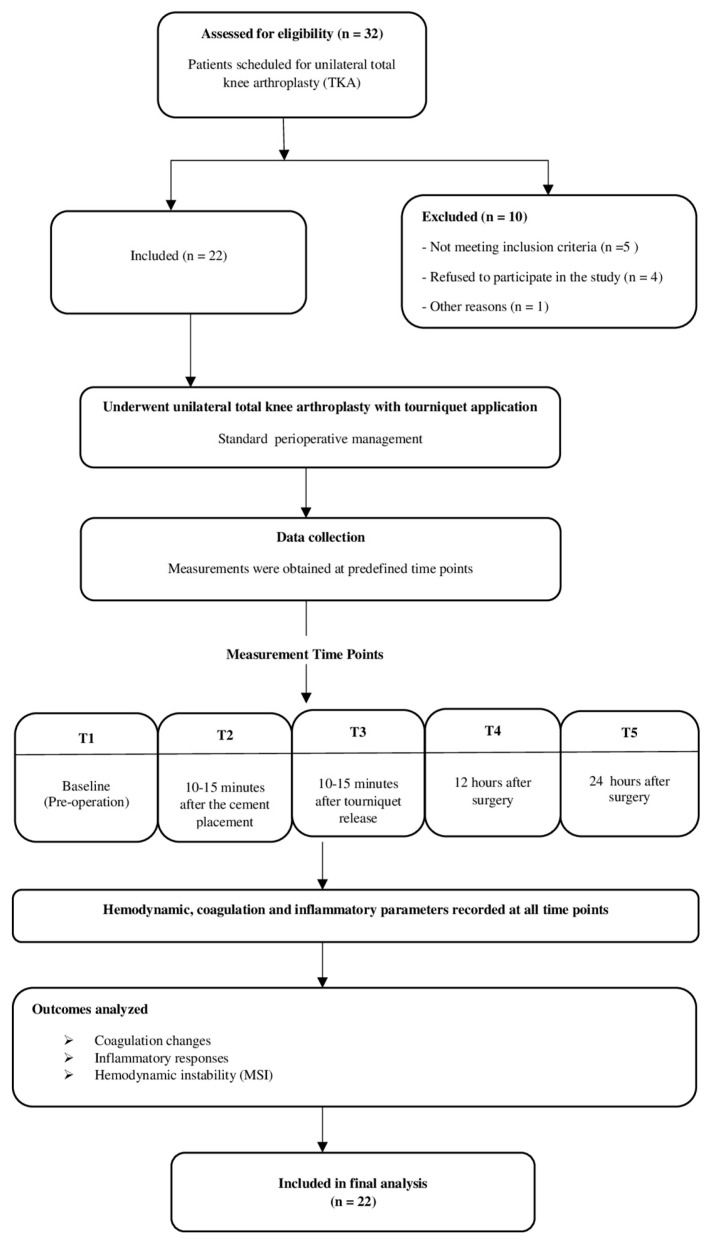
Study flow diagram and timeline of perioperative measurements. Patients scheduled for unilateral total knee arthroplasty (TKA) with tourniquet application were screened and followed prospectively. Measurements were obtained at five predefined time points: T1 (preoperative baseline), T2 (10–15 min after cement placement), T3 (10–15 min after tourniquet release), T4 (12 h postoperatively), and T5 (24 h postoperatively). Hemodynamic, coagulation, and inflammatory parameters were recorded at all time points. Outcomes included temporal changes in coagulation and inflammatory markers and their association with perioperative hemodynamic instability. MSI, modified shock index.

This prospective observational cohort study was conducted between June and August 2025 at a tertiary referral center. Ethical approval was obtained from the local institutional ethics committee (Approval No. 67926; 28 February 2023), and all study procedures complied with the principles of the Declaration of Helsinki. The study protocol was prospectively registered at ClinicalTrials.gov (NCT07002086; 2 June 2025), https://clinicaltrials.gov/study/NCT07002086 (accessed on 6 July 2026), and patient enrollment commenced after trial registration (study start date: 4 June 2025). The study was reported according to the STROBE statement. Written informed consent was obtained from all participants before enrollment. A total of 22 patients aged 65–90 years were included in the final analysis. The patient selection process is illustrated in Figure 1.

### 2.2. Inclusion and Exclusion Criteria

Inclusion criteria included ASA physical status I–III and a diagnosis of primary gonarthrosis requiring unilateral total knee arthroplasty.

Exclusion criteria included ASA physical status IV or higher, secondary gonarthrosis, rheumatologic or peripheral vascular disease, malignancy, organ dysfunction, active infection, immunosuppressive therapy, anticoagulant treatment, metabolic or renal disorders, familial hemophagocytic syndrome, substance dependence (including smoking, alcohol, or drug use), and refusal to participate in the study.

### 2.3. Outcomes and Definitions

The primary outcome was perioperative changes in coagulation parameters after tourniquet release. Secondary outcomes included inflammatory markers and their relationship with hemodynamic instability.

MSI was calculated as heart rate divided by mean arterial pressure (HR/MAP). Patients with MSI ≥ 1.3 and hypotension (MAP < 65 mmHg) were classified as hemodynamically unstable. This threshold was based on previous studies linking elevated MSI values with impaired hemodynamic compensation and increased circulatory risk in perioperative and critically ill patients. The highest perioperative MSI value was used for analysis.

### 2.4. Anesthesia and Perioperative Management

Before surgery, all participants underwent anesthetic assessment by a senior anesthesiologist. Baseline characteristics, including patient age, sex, and BMI, were documented.

All patients received standardized combined spinal–epidural anesthesia administered by anesthesiologists blinded to the study protocol. The epidural space was accessed at the L3–4 or L4–5 interspace using the loss-of-resistance technique. Subsequently, 3–4 mL of hyperbaric bupivacaine was administered intrathecally using a 27-gauge spinal needle. An epidural catheter was then inserted and secured.

Postoperative analgesia was administered via patient-controlled analgesia (PCA) using a combination of bupivacaine (2.5 mg/mL) and fentanyl (0.05 mg/mL).

### 2.5. Surgical Procedure and Tourniquet Application

To minimize procedural variability, all surgeries were completed by the same orthopaedic surgeon using a consistent operative protocol. The operated limb was elevated and wrapped with an elastic bandage. Prior to incision, tourniquet pressure was individualized at approximately 100 mmHg above baseline systolic pressure in line with published guidelines [16,17]. Tourniquet duration, inflation pressure, and operative time were recorded for all patients. All patients underwent cemented total knee arthroplasty, and polymethylmethacrylate bone cement (OGM1A^®^, Ankara, Türkiye) was used for prosthesis fixation.

### 2.6. Blood Sampling and Laboratory Analysis

Arterial blood samples were obtained through radial artery catheterization at five predefined time points: preoperative (T1), 10–15 min after cement application (T2), 10–15 min after tourniquet release (T3), postoperative 12 h (T4), and postoperative 24 h (T5). IL-1, IL-6, IL-10, and TNF-α levels were measured using ELISA. IL-1, IL-6, and IL-10 were analyzed at T1, T2, T3, and T5, whereas TNF-α was measured at T1–T4. Standardized laboratory protocols were used throughout the analyses.

#### Biochemical Analysis

Serial serum and plasma samples were collected at five predefined time points, centrifuged at 3500 rpm for 10 min, and stored at −80 °C until analysis.

Serum IL-1, IL-6, IL-10, and TNF-α levels were measured using commercial sandwich ELISA kits (BT LAB, Shanghai, China) according to the manufacturer’s instructions. All cytokines were analyzed at the first three time points; only TNF-α was measured at the fourth time point, whereas only interleukins were analyzed at the fifth.

Absorbance was measured at 450 nm, and cytokine concentrations were calculated using a four-parameter logistic (4-PL) model. All samples were analyzed in duplicate, with acceptable intra- and inter-assay variability.

To assess the balance between pro-inflammatory and anti-inflammatory responses, a pro-/anti-inflammatory ratio was calculated for each patient using the formula (Peak IL-6 + Peak TNF-α)/Peak IL-10. Higher values indicate a relative predominance of pro-inflammatory activity over anti-inflammatory cytokine responses.

### 2.7. Sample Size Calculation

The required sample size was estimated using G*Power software (version 3.1.9.4; Kiel University, Kiel, Germany) [18]. Calculations were based on a one-sided alpha level of 0.05, statistical power of 95%, and an effect size of 0.84. Reference values for perioperative PT changes were derived from a previously published study reporting preoperative and postoperative PT measurements [19]. Based on these assumptions, enrollment of at least 17 patients was considered sufficient for the analysis.

### 2.8. Statistical Analysis

Statistical evaluation was carried out using SPSS software version 16.0 (SPSS Inc., Chicago, IL, USA). Distribution normality was assessed with the Kolmogorov–Smirnov test. Because the variables did not demonstrate normal distribution, non-parametric methods were preferred throughout the analysis. Quantitative data are presented as median and interquartile range (IQR).

Temporal changes across repeated measurements were examined using the Friedman test. When overall significance was identified, post hoc pairwise comparisons were conducted with the Wilcoxon signed-rank test applying Bonferroni adjustment. Differences between hemodynamically stable and unstable patients were evaluated using the Mann–Whitney U test. Associations between variables were explored with Spearman correlation analysis.

Receiver operating characteristic (ROC) analysis was used to assess the discriminative performance of selected biomarkers for hemodynamic instability. Sensitivity, specificity, optimal threshold values, and area under the curve (AUC) were calculated. Statistical significance was defined as *p* < 0.05.

## 3. Results

### 3.1. Patient Characteristics

The study flow and patient selection process are shown in Figure 1. Baseline demographic and perioperative characteristics of the study population are summarized in Table 1.

### 3.2. Coagulation Parameters

D-dimer levels changed significantly over time (χ^2^ = 75.745, df = 4, *p* < 0.001), increasing after tourniquet release (T1 vs. T3, *p* = 0.001) and remaining elevated at 24 h (T1 vs. T5, *p* < 0.001). INR values showed a similar pattern, with significant increases both after release and at 24 h (χ^2^ = 47.861, df = 4, *p* < 0.001).

**Figure 2:**
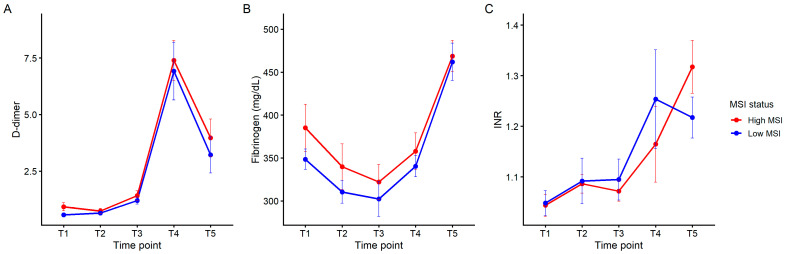
Temporal changes in coagulation parameters according to modified shock index (MSI) status. Panels display D-dimer (**A**), fibrinogen (**B**), and INR (**C**) values across the perioperative period. Data points represent mean values, and error bars indicate the standard error of the mean (SEM). Patients were classified as hemodynamically stable (*n* = 17) or unstable (*n* = 5) according to maximum MSI. T1 = preoperative baseline; T2 = 10–15 min after cement placement; T3 = 10–15 min after tourniquet release; T4 = 12 h postoperatively; T5 = 24 h postoperatively.

APTT also varied significantly over time (χ^2^ = 23.320, df = 4, *p* < 0.001). Although prolongation was evident immediately after tourniquet release (T1 vs. T3, *p* = 0.001), values returned to baseline by 24 h (T1 vs. T5, *p* = 0.258), suggesting a transient response (Table 2, Figure 2).

Figure 2 illustrates the temporal evolution of coagulation parameters according to modified shock index (MSI) status. Although differences in temporal patterns were observed visually, no statistically significant between-group differences were identified for the coagulation parameters analyzed. Therefore, the findings presented in Figure 2 should be interpreted descriptively.

**Figure 3:**
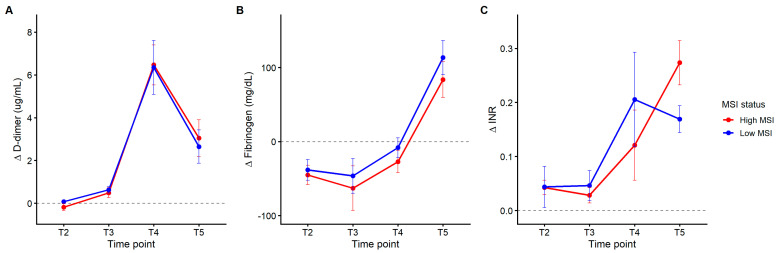
Temporal changes from baseline (Δ values) in coagulation parameters according to modified shock index (MSI) status. Panels show ΔD-dimer (**A**), ΔFibrinogen (**B**), and ΔINR (**C**), calculated relative to preoperative baseline values (T1). Data points represent mean Δ values, and error bars indicate the standard error of the mean (SEM). Patients were classified as hemodynamically stable (*n* = 17) or unstable (*n* = 5) according to maximum MSI. T1 = preoperative baseline; T2 = 10–15 min after cement placement; T3 = 10–15 min after tourniquet release; T4 = 12 h postoperatively; T5 = 24 h postoperatively.

Δ analysis provided a descriptive assessment of temporal changes from baseline. Formal between-group comparisons demonstrated no statistically significant differences in ΔD-dimer (Mann–Whitney U = 38.0, *p* = 0.724), ΔFibrinogen (U = 41.0, *p* = 0.906), or ΔINR (U = 34.0, *p* = 0.503) between hemodynamically stable and unstable patients (Supplementary Table S2). Accordingly, the patterns shown in Figure 3 should be interpreted as descriptive rather than inferential findings (Figure 3; Supplementary Table S2).

#### Cytokine Analysis

**Figure 4:**
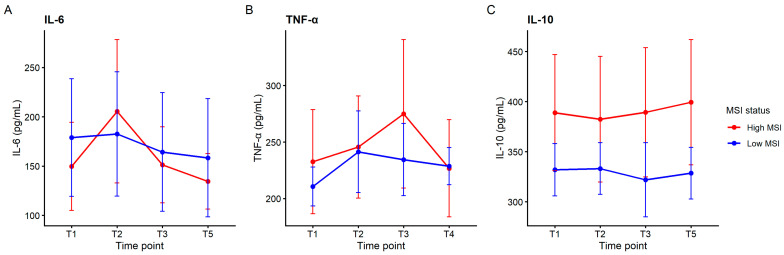
Temporal changes in inflammatory cytokine levels according to modified shock index (MSI) status. Panels display IL-6 (**A**), TNF-α (**B**), and IL-10 (**C**) concentrations measured at predefined perioperative time points. Data points represent mean values, and error bars indicate the standard error of the mean (SEM). Patients were classified as hemodynamically stable (*n* = 17) or unstable (*n* = 5) according to maximum MSI. T1 = preoperative baseline; T2 = 10–15 min after cement placement; T3 = 10–15 min after tourniquet release; T4 = 12 h postoperatively; T5 = 24 h postoperatively.

ΔPeak IL-6 levels were higher in the hemodynamically unstable group compared to the stable group; however, this difference did not reach statistical significance (Mann–Whitney U = 21.5, Z = −1.662, *p* = 0.097). No significant differences were observed between groups for ΔPeak TNF (U = 30.5, *p* = 0.346) or ΔPeak IL-10 (U = 42.0, *p* = 0.968). Similarly, the pro-/anti-inflammatory ratio did not differ significantly between groups (U = 40.0, *p* = 0.845) (Table 3, Figure 4).

### 3.3. Systemic Inflammatory Indices

Systemic inflammatory indices demonstrated significant changes over time (all *p* < 0.001, Friedman test).

The neutrophil-to-lymphocyte ratio (NLR) increased significantly following tourniquet release (T1 vs. T3, Z = −3.555, *p* < 0.001) and showed a further increase at 24 h (T1 vs. T5, Z = −4.107, *p* < 0.001).

C-reactive protein (CRP) did not show a significant change immediately after tourniquet release (T1 vs. T3, *p* = 0.407), but demonstrated a significant increase at 24 h (T1 vs. T5, Z = −4.107, *p* < 0.001).

Similarly, the systemic immune-inflammation index (SII) increased both after tourniquet release (T1 vs. T3, Z = −1.997, *p* = 0.046) and at 24 h (T1 vs. T5, Z = −4.074, *p* < 0.001). The temporal evolution of systemic inflammatory indices is summarized in Table 4.

### 3.4. Correlation Analysis

A moderate positive correlation was observed between ΔPeak IL-6 and the maximum modified shock index (MSI) (Spearman’s rho = 0.363), although this did not reach statistical significance (*p* = 0.097).

No significant correlations were found between the ΔPeak TNF and MSI (rho = 0.206, *p* = 0.358) or between the ΔPeak IL-10 and MSI.

Correlation analysis further demonstrated clustering between inflammatory cytokines and coagulation parameters. Strong positive correlations were observed among cytokines, while moderate associations were noted between inflammatory and coagulation markers (Supplementary Table S1 and Figure S1).

Additional exploratory Spearman correlation analyses were performed to evaluate the relationship between tourniquet-related variables and inflammatory and hemodynamic responses. Neither tourniquet duration nor tourniquet inflation pressure demonstrated significant correlations with ΔPeak IL-6, ΔPeak TNF-α, ΔPeak IL-10, or the maximum MSI (all *p* > 0.05). However, these exploratory findings should be interpreted cautiously given the limited sample size.

### 3.5. ROC Analysis

Receiver operating characteristic (ROC) curve analysis demonstrated that ΔPeak IL-6 had the highest discriminative performance among the evaluated biomarkers for identifying hemodynamic instability (Table 5). The optimal cut-off value, determined using Youden’s index, was 50.7, yielding a sensitivity of 80% and a specificity of 76%.

In contrast, the NLR at 24 h, SII at 24 h, and CAI demonstrated poor discriminative performance for identifying hemodynamic instability. The AUC values were 0.471 for the NLR, 0.529 for SII, and 0.482 for CAI. Although the SII demonstrated high sensitivity (80%), its specificity remained low (35%). Similarly, the CAI showed limited discrimination with a sensitivity of 60% and specificity of 53%. Overall, none of these indices demonstrated clinically meaningful predictive performance in this cohort.

The optimal cut-off values were determined using Youden’s index.

These exploratory findings suggest that ΔPeak IL-6 may have greater discriminative potential than routinely available inflammatory indices; however, its predictive performance requires validation in larger cohorts. While ΔPeak IL-6 demonstrated the most promising discriminative profile, the absence of statistically significant ROC results suggests that these findings remain preliminary and require confirmation in larger patient populations.

## 4. Discussion

This study demonstrated that tourniquet release during TKA induces significant coagulation activation and a dynamic inflammatory response, characterized by sustained increases in D-dimer and INR and transient changes in APTT. Inflammatory markers also showed distinct temporal patterns, with early increases in the NLR and SII and a delayed rise in CRP. Although no inflammatory marker was significantly associated with hemodynamic instability, ΔPeak IL-6 showed a tendency toward higher values in unstable patients and may warrant further investigation in larger prospective studies.

The increase in D-dimer and INR observed in this study supports the hypothesis that tourniquet release induces a systemic coagulation–fibrinolysis response. Watanabe et al. previously showed that coagulation and fibrinolysis markers change dynamically after TKA, supporting the presence of postoperative coagulation activation in this setting. Our findings extend this concept by showing that these changes occur early after tourniquet release and persist into the postoperative period [20].

Tourniquet-related ischemia–reperfusion injury may explain these coagulation changes. Previous studies have suggested that tourniquet use during TKA may increase leukocyte activation, endothelial injury, inflammatory response, and coagulation activity. These mechanisms are biologically consistent with the increases in D-dimer and INR observed in the present cohort [21].

Whereas D-dimer and INR showed sustained increases, APTT changes appeared transient and may reflect only temporary disruption of intrinsic coagulation pathways. These findings suggest that tourniquet release induces an early coagulation disturbance while fibrinolytic activation persists beyond the immediate reperfusion phase [22,23].

Δ analysis provided additional insight into the temporal evolution of these changes. While absolute values demonstrated overall increases, change-from-baseline analysis revealed that the magnitude and trajectory of coagulation activation differed between MSI groups. Although change-from-baseline analyses suggested differences in temporal trajectories between MSI groups, formal between-group comparisons were not statistically significant. Therefore, these observations should be interpreted as descriptive and hypothesis-generating. The correlation heatmap (Supplementary Figure S1) further suggests coordinated associations between inflammatory and coagulation pathways. The clustering of cytokines and coagulation markers indicates that these systems may not act independently but rather reflect coordinated biological responses to ischemia–reperfusion injury [24,25].

Temporal changes became more evident over time, suggesting that longitudinal analysis may better reflect postoperative physiological responses than static comparisons alone. The NLR and SII increased both after tourniquet release and at 24 h, whereas CRP elevation was mainly observed at 24 h, consistent with the delayed acute-phase nature of CRP [26,27]. Kim et al. also reported that perioperative factors may influence postoperative inflammatory responses and CRP dynamics after TKA [21].

Among cytokines, ΔPeak IL-6 appeared to be the most clinically relevant marker [28]. Although differences between stable and unstable patients did not reach statistical significance, IL-6 levels remained consistently higher in patients with hemodynamic instability. ROC analysis further suggested acceptable predictive performance for ΔPeak IL-6 (AUC = 0.747), with 80% sensitivity and 76% specificity at the selected threshold [29]. Similar postoperative IL-6 elevations following tourniquet-related skeletal muscle injury during TKA have also been reported previously [30].

ΔPeak IL-6 showed better discriminative performance than the NLR, SII, and CAI. While these routinely available inflammatory indices changed significantly over time following tourniquet release, their ROC performance remained poor, with AUC values close to 0.50. This finding suggests that although systemic inflammatory indices may reflect the magnitude of postoperative inflammation, they may not accurately identify patients at risk of hemodynamic instability. Although NLR increased significantly during the postoperative period, its ability to discriminate between hemodynamically stable and unstable patients was poor (AUC = 0.471), suggesting that NLR reflects postoperative inflammatory activation rather than the risk of hemodynamic instability. In contrast, IL-6 may more directly reflect reperfusion-related endothelial and inflammatory responses [24,31]. Increased IL-6 activity has been linked to nitric oxide dysregulation, altered vascular tone, and autonomic imbalance, all of which may contribute to perioperative hypotension and circulatory instability. These mechanisms may explain why IL-6 demonstrated greater discriminative performance than the evaluated hematologic inflammatory indices. Interestingly, despite significant postoperative increases in the NLR and SII, these indices failed to discriminate between stable and unstable patients. This observation suggests that hemodynamic instability may be more closely related to specific cytokine-mediated pathways than to generalized systemic inflammatory activation.

Bone cement–related factors may also contribute to inflammatory and hemodynamic responses after total knee arthroplasty. Previous studies have reported increased IL-6 and TNF-α levels following cemented arthroplasty [32]. Possible mechanisms include thermal effects during polymerization, monomer-related biological activity, and immune activation [11]. Because tourniquet-induced ischemia–reperfusion may also stimulate cytokine release, the inflammatory response likely reflects combined effects of surgical stress, reperfusion injury, and cement-related factors [29,30]. Although all procedures used a standardized cemented technique, material-related effects may still have influenced cytokine responses. Further studies are needed to clarify the contribution of cement-related factors to postoperative inflammatory and hemodynamic changes.

Hemodynamic instability following tourniquet release appears to involve multiple interacting mechanisms, including reperfusion injury, endothelial dysfunction, inflammatory activation, autonomic responses, and perioperative hemodynamic reserve [24,25,33]. Studies evaluating gradual or intermittent tourniquet deflation further support the contribution of tourniquet release to perioperative cardiovascular instability [33,34]. Because the MSI incorporates both heart rate and perfusion pressure, it may provide a more sensitive indicator of circulatory instability than hypotension alone. Tourniquet-related variables were generally similar across patients, limiting potential confounding effects. Additional exploratory correlation analyses demonstrated no significant associations between tourniquet duration or inflation pressure and ΔPeak IL-6, ΔPeak TNF-α, ΔPeak IL-10, or the maximum MSI (all *p* > 0.05). These findings suggest that tourniquet-related inflammatory and hemodynamic responses were not significantly influenced by tourniquet duration or inflation pressure within the range observed in the present cohort.

Several limitations should be considered when interpreting these findings. First, the sample size was relatively small, particularly in the hemodynamically unstable subgroup, which included only five patients. Consequently, the study may have been underpowered to detect significant associations for some biomarkers, increasing the risk of type II errors. Second, the observational single-center design limits causal inference and may reduce the generalizability of the findings. In addition, because all patients underwent the same surgical procedure with tourniquet application and no parallel control group without tourniquet use was included, the relative contribution of tourniquet-related ischemia–reperfusion injury versus the surgical intervention itself cannot be fully distinguished. Third, cytokine measurements were performed at predefined perioperative time points and may not have fully captured the peak inflammatory response, particularly for IL-6 and other rapidly changing cytokines. Therefore, the observed cytokine profiles should be interpreted as representative of the selected sampling intervals rather than the complete temporal inflammatory trajectory. Fourth, the study was powered based on perioperative coagulation changes rather than biomarker prediction analyses. Accordingly, the ROC analyses should be considered exploratory and hypothesis-generating. The predictive performance estimates, particularly for ΔPeak IL-6, require validation in larger cohorts with a greater number of hemodynamic instability events. Finally, hemodynamic instability was defined using a modified shock index (MSI)-based classification. Although this approach was supported by previous literature, no universally accepted threshold exists for tourniquet-related instability in total knee arthroplasty, and alternative definitions might yield different results. Despite these limitations, the study has several important strengths. The prospective design, repeated perioperative measurements, standardized anesthetic and surgical management, and the combined evaluation of coagulation parameters, cytokines, and systemic inflammatory indices provide a comprehensive assessment of physiological responses following tourniquet release.

Overall, the present findings suggest a potential interaction between inflammatory and coagulation pathways following tourniquet release during total knee arthroplasty. Although ΔPeak IL-6 showed a tendency toward association with hemodynamic instability and demonstrated moderate discriminative performance in exploratory analyses, no definitive biomarker of instability was identified. Larger multicenter studies are needed to validate these observations and clarify their clinical significance.

## 5. Conclusions

Tourniquet release during total knee arthroplasty triggers both coagulation activation and inflammatory responses, highlighting the multifactorial nature of reperfusion-related physiological changes. While conventional inflammatory indices including the NLR, SII, and CAI showed poor discriminative performance for identifying hemodynamic instability, ΔPeak IL-6 demonstrated the highest AUC among the evaluated markers and may warrant further investigation as a potential biomarker of perioperative hemodynamic instability. From a clinical perspective, these findings suggest that cytokine-mediated inflammatory responses may contribute to interindividual variability in hemodynamic responses following tourniquet release. Improved understanding of these pathways may support future risk-stratification strategies and facilitate the identification of patients who may benefit from closer perioperative hemodynamic monitoring. Given the exploratory nature of this study and the limited sample size, larger multicenter studies are needed to validate these findings and further clarify the relationship between inflammatory activation, coagulation disturbances, and perioperative hemodynamic instability.

## Figures and Tables

**Table 1:** Demographic and perioperative characteristics of the study population (*n* = 22).

Variable	Value
Age (years)	68.50 (64.00–72.25)
BMI (kg/m^2^)	35.58 (32.22–38.28)
Sex, *n* (%)	
Women	18 (81.8)
Men	4 (18.2)
ASA physical status, *n* (%)	
ASA II	20 (90.9)
ASA III	2 (9.1)
Tourniquet duration (min)	90.00 (80.25–126.00)
Tourniquet pressure (mmHg)	300.00 (300.00–320.00)

Values are presented as median (interquartile range) or number of patients (percentage). BMI, body mass index; ASA, American Society of Anesthesiologists.

**Table 2:** Coagulation Parameters Over Time.

Variable	T1 (Baseline)	T3 (Post-Release)	T5 (24 h)	*p* (Friedman)
D-dimer	0.69 (0.52–0.85)	1.18 (0.94–1.53)	2.78 (1.92–3.43)	<0.001
INR	1.05 (0.98–1.11)	1.08 (1.03–1.12)	1.27 (1.17–1.34)	<0.001
APTT	26.40 (25.30–27.40)	24.35 (21.90–26.40)	26.40 (25.80–29.20)	<0.001

Changes in coagulation parameters over time. D-dimer and INR levels increased significantly following tourniquet release and remained elevated at 24 h, whereas APTT showed a transient prolongation at T3 with no significant difference at 24 h. Values are presented as median (interquartile range). Values are presented as median (interquartile range, IQR). *p* values shown in the final column represent the overall time effect across all measurement time points (T1, T3, and T5), as assessed using the Friedman test for repeated measures. Post hoc pairwise comparisons between individual time points were performed using Wilcoxon signed-rank tests and are reported separately in Section 3. INR, international normalized ratio; APTT, activated partial thromboplastin time.

**Table 3:** Cytokine Comparison Between Groups.

Variable	Stable (*n* = 17)	Unstable (*n* = 5)	*p*-Value
ΔPeak IL-6	12.26 (0.00–51.80)	73.77 (29.39–333.95)	0.097
ΔPeak TNF	57.83 (21.95–77.83)	116.90 (21.54–177.32)	0.346
ΔPeak IL-10	26.98 (0.00–77.60)	27.49 (6.65–62.73)	0.968
Pro-/Anti-inflammatory ratio	1.06 (0.94–1.22)	1.05 (0.91–2.03)	0.845

Comparison of cytokine responses between hemodynamically stable and unstable patients. ΔPeak IL-6 showed a trend toward higher values in the unstable group, whereas TNF, IL-10, and the pro-/anti-inflammatory ratio did not differ significantly. Values are presented as median (interquartile range). ΔPeak represents the change from baseline (preoperative) to the maximum observed value across perioperative time points. IL-6, interleukin-6; TNF, tumor necrosis factor-alpha; IL-10, interleukin-10.

**Table 4:** Inflammatory Indices Over Time.

Variable	T1	T3	T5	*p* (Friedman)
**NLR**	1.78 (1.28–2.26)	2.69 (1.87–3.81)	5.55 (3.14–7.57)	<0.001
**CRP**	3.65 (2.38–5.23)	3.55 (2.28–4.75)	140.40 (101.43–167.53)	<0.001
**SII**	492.75 (357.77–630.52)	579.00 (410.25–807.50)	1196.15 (758.59–1643.31)	<0.001
**PLR**	112.00 (90.14–131.87)	131.24 (106.52–172.41)	154.17 (111.20–196.86)	<0.001
**AISI**	201.43 (127.30–271.42)	233.01 (114.88–400.93)	888.75 (547.84–1469.27)	<0.001
**Procalcitonin**	0.04 (0.04–0.05)	0.04 (0.03–0.05)	0.25 (0.18–0.53)	<0.001

Temporal changes in systemic inflammatory indices. NLR, SII, PLR, and AISI increased significantly both after tourniquet release and at 24 h, whereas CRP and procalcitonin demonstrated a delayed increase at 24 h. Values are presented as median (interquartile range). *p* values were calculated using the Friedman test for repeated measures. NLR, neutrophil-to-lymphocyte ratio; SII, systemic immune-inflammation index; PLR, platelet-to-lymphocyte ratio; AISI, aggregate index of systemic inflammation; CRP, C-reactive protein.

**Table 5:** ROC analysis of inflammatory biomarkers for hemodynamic instability.

Variable	AUC (95% CI)	*p* Value	Cut-Off	Sensitivity	Specificity
ΔPeak IL-6	0.747 (0.452–1.000)	0.100	50.7	80	76
NLR (T5)	0.471 (0.174–0.767)	0.845	5.55	20	41
SII (T5)	0.529 (0.250–0.809)	0.845	932.7	80	35
CAI	0.482 (0.182–0.783)	0.906	0.22	60	53

Receiver operating characteristic (ROC) curve analysis evaluating the discriminative performance of selected biomarkers for hemodynamic instability. Area under the curve (AUC), 95% confidence intervals (CIs), optimal cut-off values, sensitivity, and specificity are reported. Optimal thresholds were identified using Youden’s index. AUC, area under the curve; CI, confidence interval; IL-6, interleukin-6; NLR, neutrophil-to-lymphocyte ratio; SII, systemic immune-inflammation index; CAI, cytokine–coagulation activation index.

## Data Availability

Data available on request from the authors.

## Supplementary Materials

The following supporting information can be downloaded at https://www.mdpi.com/article/10.3390/jcm15145386/s1, Figure S1: correlation heatmap of inflammatory cytokines and coagulation parameters; Table S1: correlation analysis; Table S2: between-group comparisons of changes from baseline [Δ values] in coagulation parameters according to hemodynamic stability status, and the STROBE Checklist.
