# Supplementary material for: Coagulation and Inflammatory Responses After Tourniquet Release in Total Knee Arthroplasty: Association with Hemodynamic Instability
Source: J Clin Med. 2026 Jul 9;15(14):5386. doi: 10.3390/jcm15145386 (PMC13412553; doi:10.3390/jcm15145386)
Supplement: Supplementary file 1 [file jcm-15-05386-s001.zip › Supplementary Table S1.pdf]

**Supplementary Table S1.** Correlation Analysis

| **Variable** | **Spearman's rho** | ***P*-value** |
|---|---|---|
| ΔPeak IL-6 vs MSI | 0.363 | 0.097 |
| ΔPeak TNF vs MSI | 0.206 | 0.358 |
| ΔPeak IL-10 vs MSI | 0.009 | 0.969 |

Correlation between inflammatory markers and hemodynamic instability. A moderate positive correlation was observed between ΔPeak IL-6 and maximum modified shock index (MSI), although this did not reach statistical significance. No significant correlations were found for TNF or IL-10.

ΔPeak indicates the difference between the peak value and baseline (preoperative) level.

MSI, modified shock index (heart rate/mean arterial pressure); IL-6, interleukin-6; TNF, tumor necrosis factor-alpha; IL-10, interleukin-10.
