# Supplementary material for: Coagulation and Inflammatory Responses After Tourniquet Release in Total Knee Arthroplasty: Association with Hemodynamic Instability
Source: J Clin Med. 2026 Jul 9;15(14):5386. doi: 10.3390/jcm15145386 (PMC13412553; doi:10.3390/jcm15145386)
Supplement: Supplementary file 1 [file jcm-15-05386-s001.zip › Supplementary Table S2.pdf]

Supplementary Table S2. Between-group comparisons of changes from baseline (Δ values) in coagulation parameters according to hemodynamic stability status.

Variable	Stable (n = 17) Median (IQR)	Unstable (n = 5) Median (IQR)	Mann–Whitney U	P-value
**ΔD-dimer**	0.51 (0.94)	0.51 (0.56)	38.0	0.724
**ΔFibrinogen**	-33.0 (84.5)	-20.0 (182.5)	41.0	0.906
**ΔINR**	0.03 (0.08)	0.01 (0.09)	34.0	0.503

Values are presented as median (interquartile range, IQR). Δ values represent the change from baseline (T1). Patients were classified as hemodynamically stable or unstable according to maximum modified shock index (MSI).
