# Supplementary figures and images for: Coagulation and Inflammatory Responses After Tourniquet Release in Total Knee Arthroplasty: Association with Hemodynamic Instability
Source: J Clin Med. 2026 Jul 9;15(14):5386. doi: 10.3390/jcm15145386 (PMC13412553; doi:10.3390/jcm15145386)

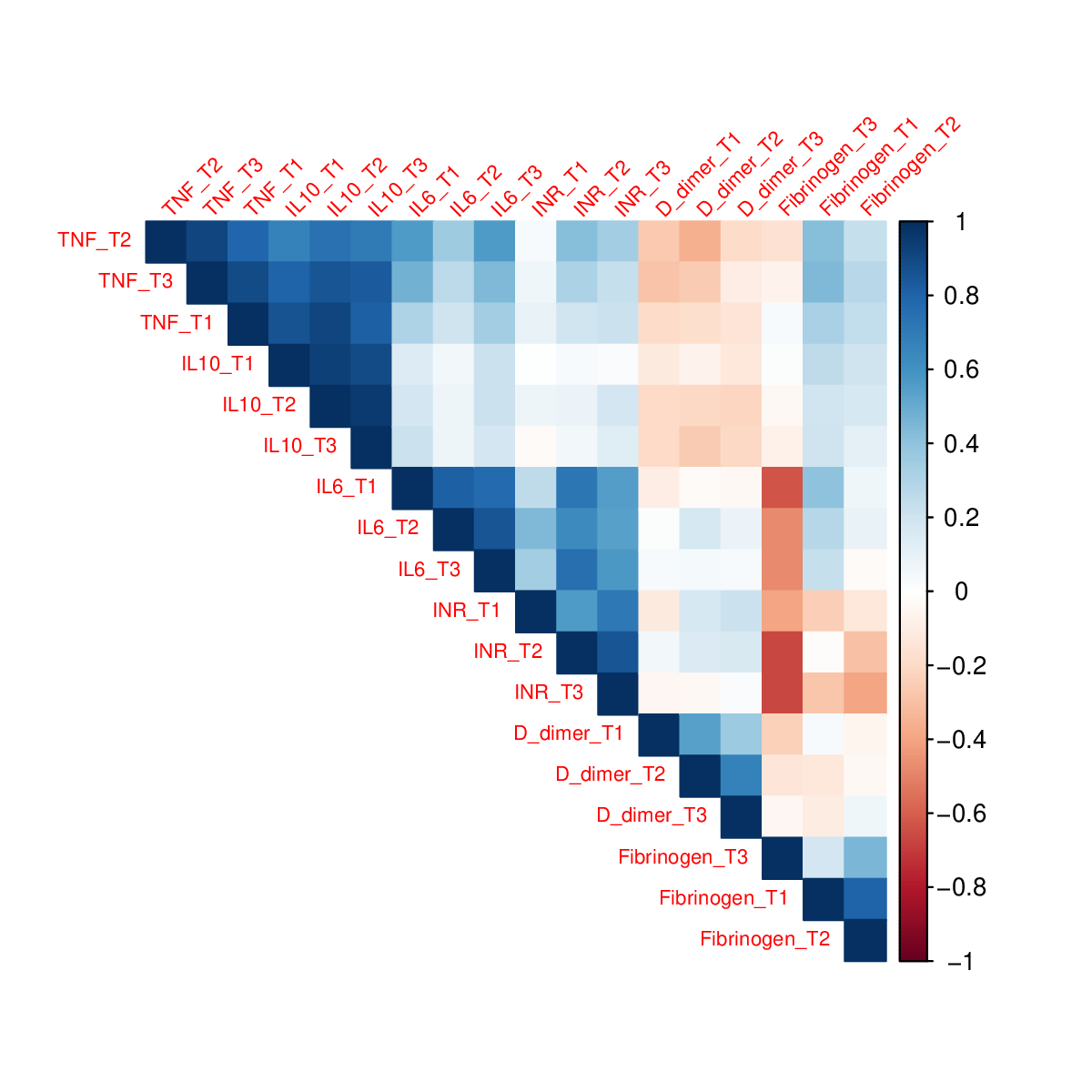

Supplement: Supplementary file 1 [file jcm-15-05386-s001.zip › Supplementary Figure S1.tiff]
